# The Effect of Berry Pomace on Quality Changes of Beef Patties during Refrigerated Storage

**DOI:** 10.3390/foods11152180

**Published:** 2022-07-22

**Authors:** Živilė Tarasevičienė, Indrė Čechovičienė, Aurelija Paulauskienė, Milda Gumbytė, Aušra Blinstrubienė, Natalija Burbulis

**Affiliations:** 1 Department of Plants Biology and Food Science, Faculty of Agronomy, Agriculture Academy Vytautas Magnus University, Donelaicio Str. 52, LT-44248 Kaunas, Lithuania; indre.cechoviciene@stud.vdu.lt (I.Č.); aurelija.paulauskiene@vdu.lt (A.P.); 2 Laboratory of Chemical and Biochemical Research for Environmental Technology, Department of Environment and Ecology, Faculty of Forest Sciences and Ecology, Agriculture Academy Vytautas Magnus University, Donelaicio Str. 52, LT-44248 Kaunas, Lithuania; milda.gumbyte@vdu.lt (M.G.); ausra.blinstrubiene@vdu.lt (A.B.); natalija.burbulis@vdu.lt (N.B.)

**Keywords:** berry press cake, fatty acids, meat quality, organic volatile compounds

## Abstract

This study aims to evaluate the ability of raspberry and blackberry pomace to inhibit lipid oxidation and prolong the refrigerated storage of beef patties. Berry pomace was incorporated into beef patties at the concentration of 1, 3, and 5%. Packed patties were stored for 9 days at 4 °C temperature and the quality of the meat was evaluated on the 0, 3rd, 6th, and 9th day. The natural mass loss during storage, the pH as well as the lipid oxidation were evaluated by thiobarbituric acid-reactive substance (TBARS) method. GC was used to determine the amount of fatty acids and e-nose, based on ultrafast gas chromatography, was used for the determination of volatile organic compounds in beef patties before and after the storage. The highest mass loss during refrigerated storage was observed in the control beef patties, while the berry pomace absorbed water and reduced the loss. The pomace additive influenced the decrease in the patties pH during the storage. Berry pomace can be very effective in relation to lipid oxidation, and as little as 1% of berry pomace influenced the decrease in the TBAR’s values in the patties stored for nine days by 3.06 and 2.42 times, depending on the pomace compared to the control patties. The use of berry pomace in meat products can reduce lipid oxidation, increase their fiber content and act as a thickener, as well as contribute to the usage of agri-food by-products.

## 1. Introduction

Freshly ground meat is widely used all over the world as a component of various food products, and is especially important for the production of beef patties [1]. The United Nations Food and Agriculture Organization projects that the consumption of meat and meat products will increase by 73 percent by 2050, due to the increasing population. Microbial growth and lipid oxidation are two main causes of meat deterioration, and they have an influence on the nutritional value, sensory attributes, and safety of the product [2]. The consumers demand healthy and safe meat products, which encourages the use of novel processing methods and natural antioxidants [3]. Therefore, as a result scientists are looking for solutions to prolong the shelf life of minced meat, using direct or indirect (as parts or storage materials) natural antioxidants and antimicrobics from plant materials [2,3,4,5,6,7,8].

In recent years, the interest in the usage of agro-food by-products has increased, which has led to green and sustainable developments, because the management of the by-products from fruit processing is one of the major problems for the agro-fruit industries [9,10]. Usually, tons of pomace are discarded directly into the dump or alternatively utilized in animal feed production. This causes either environmental problems, or, due to insufficient protein content, such feed does not guarantee valuable diets for animals [11,12]. One of the ways to use these by-products is to extract the antioxidant compounds from berry pomace, which is rich in phenolic compounds, polysaccharides, dietary fiber, and other bioactive nutrients. In order to obtain the berry extract, traditional extraction methods can be used, as well as novel green technologies [9]. On the other hand, berry pomace is composed of diverse parts: skins; pressed flesh; and seeds, which contain not only lipids and hydrophilic antioxidants, such as polyphenols, but also dietary fiber [9,13]. The dietary fiber, as well as the phenols, can increase the shelf life, freshness, and textural properties of products. Food products with a high amount of fiber have been reported as being beneficial for human health [14]. Therefore, the usage of the berry pomace, rather than the extract, can be more beneficial.

The *Rubus* L. berries are very popular because of their flavor, high nutritional value, and wide applicability. Nowadays, red raspberries, as well as blackberries, are processed and sold as juices, syrups, jellies, other processed products, and seed oil, but the by-product, pomace, is neglected in most cases [15]. The Rubus raspberries and blackberries contain a large variety of components, such as fructose, glucose, protein, minerals (K, Ca, Mg, Fe, Zn, and Mn), ascorbic acid, carotenoids, linoleic acid, and phenolics (phenolic acids, anthocyanins, and other flavonoids) [16,17].

Raspberry pomace contains about 77.5% dietary fiber, out of which 75% is insoluble dietary fiber and 2.5% is soluble dietary fiber, while the total dietary fiber values in blackberries are around 79.60%, out of which 74.55% is insoluble and 5.05% is soluble [18,19]. The phenol compounds, as well as fiber, are abundant in *Rubus* L. berries’ pomace. The total amount of bound phenolic compounds in the extract of blackberry pomace was 10.44 mg g^−1^ dry weight, with 12 polyphenols identified [20]. The raspberry pomace without seeds contains about 24.14 mg GAE g^−1^ dry matter of total phenols and raspberry seeds—25.40 mg GAE g^−1^ [21].

Recent research found that the extracts of berries’ pomace has a positive influence on the shelf life and lipid oxidation of meat, substituting for synthetic antioxidants and preservatives as well as presenting potential functional properties for consumers. According to Basanta et al. [22], the extract of Japanese plum (*Prunus salicina*) pulp and skin proved to be a natural preservative for chicken breast patties, with a positive effect on texture (springiness) as well as maintaining a natural color. Defatted chokeberry pomace extract at the concentration of 2% inhibited the formation of oxidation products in raw pork burgers and cooked ham during storage [23].

In terms of the pomace powder’s direct incorporation into meat products, it was estimated that destoned olive cake powder had an inhibitory effect on fat oxidation and inhibited microbiological growth in beef patties [2]. In addition, Mosambi peel powder incorporated into buffalo meat sausages and patties showed a potential to be a functional ingredient because of an enhanced fiber content and the functional properties, along with improved storage stability of the meat products [24].

To our knowledge, there are limited data on the berry pomace’s influence on meat product quality; therefore, the present study is focused on an evaluation of the effects of the incorporation of raspberry and blackberry pomace in beef patties on meat quality changes during the refrigerated storage.

## 2. Materials and Methods

### 2.1. Materials

All of the chemicals used in the food analysis were of analytical grade and obtained from standard commercial suppliers.

### 2.2. Berry Pomace Preparation

The freshly pressed pomace of raspberry and blackberry was obtained from a local juice industry in Kaunas County, Lithuania. The pomace was dried in a hot-air drying convection oven (Model SLN 240, Wodzisław Śląski, Poland) at 45 °C for 24 h, and ground with a food mill (Model Retsch ZM200, Haan, Germany) to flour consistency (0.2 mm particle size).

### 2.3. Beef Patty Preparation

The ground beef (19.3% proteins, 14.5% fats) made from the beef neck was obtained from a local supplier, as well as the common salt and drinkable water used in the formulation of the beef patties. The patties were made by mixing beef with additives in a food mixer for 5 min. According to the formulations, the control patties were prepared from 920 g of meat, 15 g of salt, and 50 mL of water, and the other patties in the same formulation as the control patties, with the addition of 1, 3, and 5% berry pomace flour. Finally, patties were manually formed with a thickness between 12 and 15 mm and a weight between 100 and 105 g. The packed patties were stored for 9 days at 4 °C temperature. The analyses were performed every 3 days, which means on the 0, 3rd, 6th, and 9th storage days; only analyses of the fatty acids and volatile compounds were performed before and after the storage.

### 2.4. Measurement of pH

The beef patties’ pH value was measured by using the potentiometric method with a pH-meter Cyber Scan 1500 (Eutech Instruments, Singapore). The equipment was calibrated using two buffers’ solutions (pH = 4.0 and pH = 7.0). The measurements were performed in triplicate for each sample.

### 2.5. Total Phenolic Compounds Determination

Briefly, 2.54 g of dried berries pomace mixed with 50 mL of 70% (*v*/*v*) ethanol for 20 min. in an ultrasound bath at 40 °C. Then, the pomace was filtered and the procedure was repeated twice with 10 mL of ethanol. All of the supernatant was diluted in a 50 mL flask with ethanol. A total of 0.5 mL of the extract was mixed with 2.5 mL of Folin–Ciocalteu reagent and 2 mL of sodium carbonate solution (7.5%). The absorbance was verified in a spectrophotometer at 725 nm after 1 h of incubation in the dark at room temperature. Gallic acid was used as the standard for the calibration curve. The results were expressed in mg of gallic acid equivalent (GAE) in g of dry matter [25].

### 2.6. Total Anthocyanins

The total anthocyanins were quantified by the pH-differential method, according to Giusti and Wrolstad [26]. The berry pomace extract was prepared by mixing 2.5 g of pomace flour with ethanol (60%) in an ultrasound bath for 15 min. The extracts of the berry powders were diluted with two buffer solutions at pH 1 and 4.5, and the absorbance was measured at 520 and 700 nm. The cyanidin-3-glucoside (449.2 g mol^−1^) was used as a standard with a molar absorptivity coefficient of [L mol^−1^ cm^−1^]. The results obtained were expressed as mg of cyanidin-3-glucoside (C3GE) per 100 mg of dry matter [27].

### 2.7. Natural Mass Loss (%)

The weight loss of the beef patties was calculated from the differences in mass before and after storage, according to the formula: X = ((A − B)∙100)/A; where A is the mass of the sample before storage, g; B is the mass of the sample after storage, g.

### 2.8. Measurement of Lipid Oxidation

The lipid oxidation was evaluated by thiobarbituric acid-reactive substance (TBARS), according to Jongberg et al. [28], and expressed as mg of malonaldehyde/kg of the meat sample, which was calculated using a molar extinction coefficient (156,000 M^−1^·cm^−1^). Briefly, 5 g of the beef patties were homogenized with 15 mL of 7.5% trichloroacetic acid with 0.10% propyl gallate and 0.10% EDTA and filtered. The mixture of 5 mL of the filtrate and 20 mM thiobarbituric acid was incubated in a water bath at 100 °C for 40 min. The absorbance was read at 532 nm with a UV-Vis spectrophotometer (Spectro UV-VIS dual beam UVS-2800, JAV) [28].

### 2.9. Determination of Fatty Acids

The content of the fatty acids was determined according to LST EN ISO 15304:2003/AC:20052 [29] with gas chromatograph Shimadzu GC—2010 PLUS (Shimadzu Corporation, Kyoto, Japan) using a flame ionization detector and column BPX—70, 120 m. The meat samples were prepared according to the procedures described in LST EN ISO 12966-2: 2011 [30]. The fatty acids were methylated with anhydrous KOH in methanol.

A total of 25 ± 0.01 g of meat in a plastic test tube was mixed with 25 mL of hexane, then mixed thoroughly by vortex for 1 h. From the test tube, 4 mL of extract was mixed with 200 µL 2 mol/L KOH solution then centrifuged at the speed of rpm and left to layer for 30 min. The amount of 1 µL of the extract was taken for HPLC analysis.

The oven temperature program was as follows: 60 °C for 2 min; 20 °C min^−1^ ramp to 230 °C; and kept for 25 min. The carrier gas was nitrogen. The temperature of the evaporator was 250 °C and the detector was 270 °C. The fatty acid kit used for fatty acid identification was the “Supelco 37 Component FAME Mix”.

### 2.10. Volatile Compounds Analysis

The Heracles II electronic nose (Alpha M.O.S., Toulouse, France) based on ultrafast gas chromatography was applied to analyze the volatile compounds of beef patties with berry pomace additives, according to the method described by Wojtasik-Kalinowska et al. [31]. Briefly, 3 g of beef patties meat was placed in glass vials (20 mL) and capped with a Teflon-faced silicon rubber cap. The vials were placed in the automatic sampler. Each vial was incubated at 50 °C for 10 min under agitation (500 rpm). The carrier gas, hydrogen, was circulated at 1 mL min^−1^. The accumulated gas in the headspace was then injected into GC with 10 m length, 0.18 mm internal diameter, two different polarity columns non-polar MXT-5 (5% diphenyl) and semi-polar MXT-1701 (14% cyanopropylphenyl) with two flame ionization detectors (FID). The injected volume was 2500 µL and the injector temperature was 200 °C. The temperature of the two flame ionization detectors was 280 °C [32]. The injection on the e-nose was carried out on three replicates. The method was calibrated using an alkane solution (n-butane to n-hexadecane) in order to convert retention time into Kovats indices, and to identify the volatile compounds using the AroChemBase database.

### 2.11. Color Measurements

The surface color of the beef patties, as well as the pomace color, were detected by the Color Flex spectrophotometer (Hunter Associates Laboratory Inc., Reston, VA, USA) from four different areas on the beef surface of each patty and three beef patties per treatment, and five replicates of the berry pomace were analyzed to obtain an average value and expressed as L*, a*, and b* CIE coordinates. The L* (lightness) value ranges from 0 = black to 100 = white, the a* (redness) ranges from green (negative) to red (positive), and b* (yellowness) values are blue (negative) to yellow (positive).

### 2.12. Statistical Methods

The data obtained from three replications were analyzed by one (berry pomace data) and two-way analysis of variance (ANOVA), using the Statistica software (Statistica 12; StatSoft, Inc., Tulsa, OK, USA). The differences among the means were compared using the Fisher post-hoc test at a significance level of 0.05. The correlation coefficient between TBARS and the color of beef patties was calculated. The principal component analysis (PCA) was performed to evaluate the relationships between the applications of the berry pomace, different patties’ storage time, and the fatty acid content with XLSTAT software version 2019.3.02 (Addinsoft, Paris, France).

## 3. Results and Discussion

### 3.1. Total Phenols and Color of Berry Pomace

According to Szymanowska, Baraniak, and Bogucka-Kocka (2018), approximately one-third of the total phenolic content remains in the *R. idaeus* pomace [33]. The total phenols and anthocyanins content is shown in Table 1. The highest amount of total phenols and anthocyanins was determined in the raspberry pomace. The amount of the anthocyanins differs by 7.52 times, while the amount of total phenols differs by 1.07 times. According to Kalušević et al. [21], the research results of the blackberry pomace had a significantly higher total phenolic content (10.1 mg GAE g^−1^) and total anthocyanins content (6 mg C3GE g^−1^) compared to the raspberry pomace (8.2 and 3.6 mg g^−1^, respectively). The results obtained during the research were the opposite, and it can be concluded that the juice production process, as well as the pomace drying, species of the berries, varieties, agricultural practices, etc., influences the chemical content of the berry pomace. The lower amount of anthocyanins in the pomace may be related to the fact that these compounds are mostly transferred into the juice, and the remaining anthocyanins in pomace are degraded through the drying process [34]. Therefore, the anthocyanins content in the berry pomace was low.

**Figure 1:**
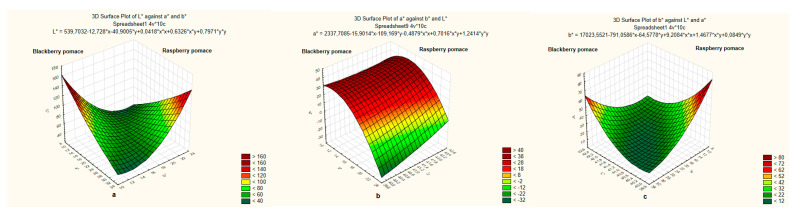
Berries pomace color L* coordinate values (**a**), a* (**b**) and b* (**c**).

All of the values of the color parameters in the berry pomace differed significantly (Figure 1). The raspberry pomace was brighter and L* value was 2.18 NBS units higher than blackberry. The main pomace color difference was observed in the a* and b* coordinates values. The raspberry pomace was redder than the blackberry, respectively, the a* values were 32.67 and 10.24 and the blackberry pomace was bluer than the raspberry, respectively, the b* values were 12.93 and 21.52 NBS units (Figure 1a–c).

### 3.2. Beef Patties Natural Mass Loss and pH

The highest mass loss during the refrigerated storage was observed in the control beef patties, while the berry pomace absorbed water and reduced the loss (Table 2). The total dietary fiber content in the raspberries was 542 g kg^−1^ dw [35] and the lignin, which is used in the food industry as the thickener, predominated [15]. Taraseviciene et al. reported that the neutral detergent fiber (cellulose, hemicellulose, and lignin) content in raspberry pomace consists of 68.20% in dry matter [36]. The blackberries contain an even higher amount of fiber, according to Sozzi, A. et al. [37] the total dietary fiber in the blackberry pomace consists of 796 g kg^−1^ dw. However, the research revealed that the berry pomace, amount of additive, and storage time did not exert a significant influence on the natural mass loss of the beef patties. By using the berry pomace powder instead of an extract, we benefited from enriching the meat product with fiber, as well as reducing mass loss during the storage.

The pH value is an important indicator of meat quality and is closely connected with protein stability and microbiological contamination [38,39]. In the Table 3 data shows that the raw beef pH was 5.41 and in line with results of another researcher, whose pH value was 5.62 [15]. The pH value of the beef patties without the pomace did not change statistically significantly, while the berry pomace influenced the decrease in the patties pH after three storage days (Table 3). A slight fluctuation was observed in the pH during the patties storage. It may be related to the fact that the berry pomace, as well as the berries, contain the organic acids, such as citric acid, malic acid, tartaric acid, and oxalic acid [40], and the release of the organic acids takes place gradually. A significant difference between the additive’s effect on beef patties pH was not observed, perhaps because the acidity of the berry pomace is similar. The amount of the predominant citric acid in the raspberry pomace, according to Górnás, P. et al. [35], is up to 48 g kg^−1^ dm, while in the blackberry pomace, the main acid is malic acid, and its amount consists of 57.06 g kg^−1^ [41]. In addition, such conditions were unfavorable for the growth of the microorganisms. Without exposure to microorganisms, malic acid can act as an antioxidant and influence lipid oxidation [42].

### 3.3. Lipids Oxidation and Fatty Acids Changes in Beef Patties during Storage

**Figure 2:**
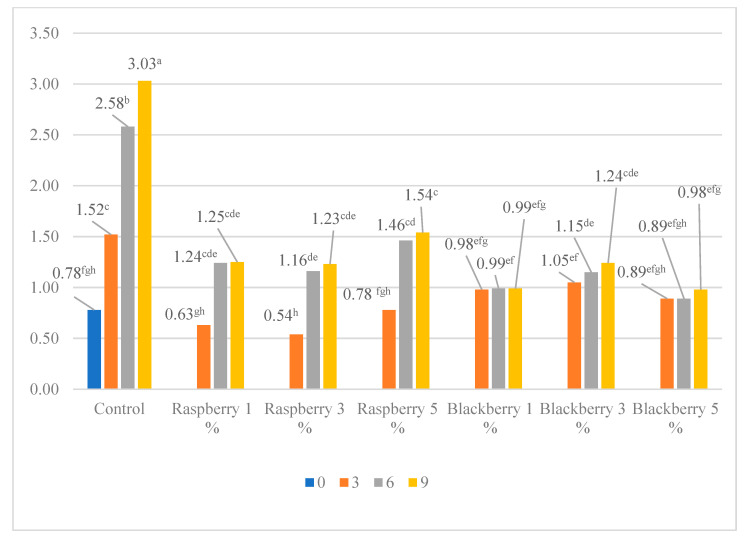
TBARS levels in beef patties stored for nine days, malondialdehyde (MDA) (mg) present in 1 kg of meat. a, b, c, e, f, g, h Values in a columns with different superscripts differ significantly at *p* ≤ 0.05.

The effect of the berry pomace additive on meat lipid oxidation is reported in Figure 2. The most intensive lipid oxidation was observed in the control beef patties stored for nine days. There were no observed differences in the TBARS levels in beef patties with additives. Even 1% of the raspberry and blackberry additive was effective in reducing the lipids’ oxidation. The difference in the TBAR’s values in the patties stored for nine days, without the pomace additive and with the additive, accounted for 1.97 to 3.09 times.

The scientific data reveal that the berries’ and fruits’ bioactive compounds, such as polyphenols (i.e., phenolic acids, flavonols, anthocyanins, tannins) and ascorbic acid may act as strong natural antioxidants, able to decrease the lipid and protein oxidation [40]. No less important, the antioxidant determined in plant tissues as well as in animals is vitamin E (tocopherols), for which the antioxidant activity is concentration-dependent [43]. According to Hendawy et al.’s [44] research data, the total tocopherol content in cold-pressed raspberry seeds’ oil is 185.1 mg 100 g^−1^ oil. Mildner-Szkudlarz et al. [45] provided data that raspberry seeds contain about 235.37 mg 100 g^−1^ oil of total tocopherols, while according to Li et al. [46], in blackberry seeds the total tocopherols are 28.63 mg 100 g^−1^ oil. According to literature sources and the data shown in Figure 2, preliminary observations can be made that the tocopherols are not the main antioxidant compounds in the berry pomace. It is most likely that the oxidative stability of the beef patties can be attributed to the phenols’ content in the berry pomace. The chokeberry pomace extract at the concentration of 2% isolated with pressurized ethanol from defatted by supercritical CO2 chokeberry pomace significantly inhibited the lipid oxidation in the pork burgers after 16 days of storage, by 2.3 times compared with the control meat [23]. Flavonoid cloudberry and beetroot extracts were shown to be effective in reducing lipid oxidation in cooked pork patties [47].

Lipid oxidation, especially accelerated by meat grinding, has a negative effect not only on the meat quality properties, such as taste, nutrition value, and texture, but also on color [48]. A correlation analysis between color parameters and TBARS showed that the control beef patties were less red, and bluer when the lipid oxidation was the most intensive, while a correlation was not observed between the lightness and TBARS level. The same tendencies were in the beef patties with raspberry pomace, except for the lightness. The beef patties with raspberry pomace became darker when the TBARS increased. Significant correlations between the TBARS and color parameters were not observed in the case of the beef patties with blackberry pomace, that might be due to the purple pigment in the blackberry pomace (Table 4). Myoglobin, as the main coloring component of the meat, is oxidized by intermediate compounds of lipid oxidation, which induce a change from the bright cherry-red color to brownish [49]. Myoglobin is found in three forms: oxymyoglobin; deoxymyoglobin; and metmyoglobin. Oxymyoglobin oxidizes into metmyoglobin during meat storage and its amount depends on the storage conditions. According to Xiaoting Wanga et al., a decrease in the beef patties’ color a* value promoted the accumulation of the metmyoglobin and a significant increase in the TBARS values (*p* < 0.05) [50].

**Figure 3:**
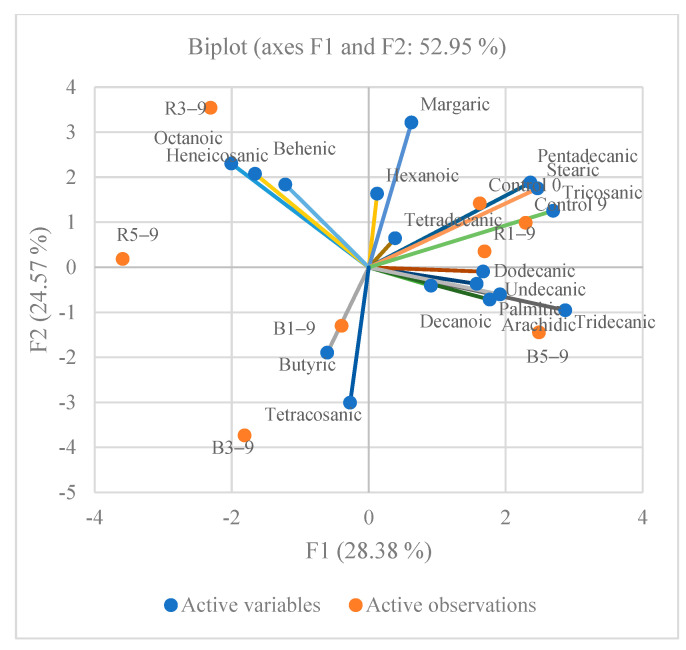
Principal component analysis (PCA) for saturated fatty acids of meat patties with different berries pomace additive stored for nine days (Control 0—meat patties without additives before storage; Control 9—without additives stored for 9 days); R1–9 and B1–9—with 1% of raspberry and blackberry pomace additives after 9 days of storage; R3–9 and B3–9—with 3% of raspberry and blackberry pomace additives after 9 days of storage; R5–9 and B5–9—with 5% of raspberry and blackberry pomace additives after 9 days of storage).

**Figure 4:**
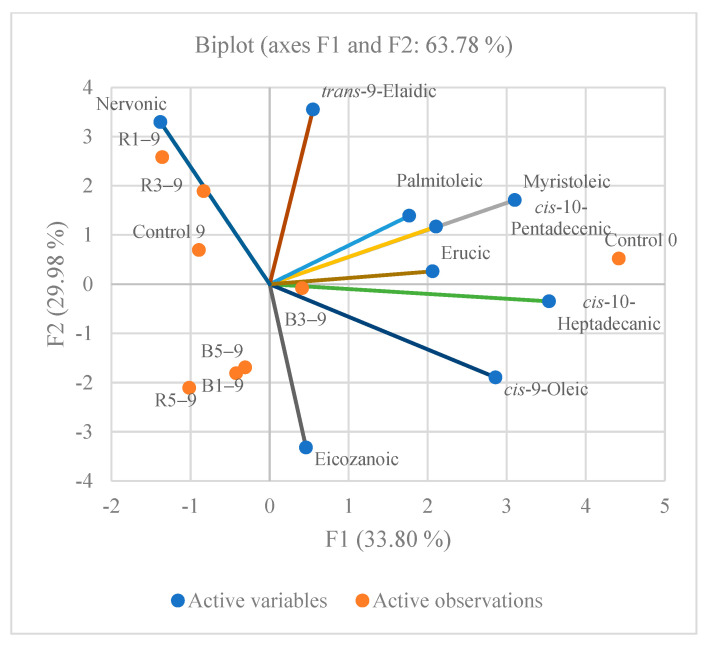
Principal component analysis (PCA) for monounsaturated fatty acids of meat patties with different berries pomace additives stored for nine days (Control 0—meat patties without additives before storage; Control 9—without additives stored for 9 days), R1–9 and B1–9—with 1% of raspberry and blackberry pomace additives after 9 days of storage; R3–9 and B3–9—with 3% of raspberry and blackberry pomace additives after 9 days of storage; R5–9 and B5–9—with 5% of raspberry and blackberry pomace additives after 9 days of storage).

**Figure 5:**
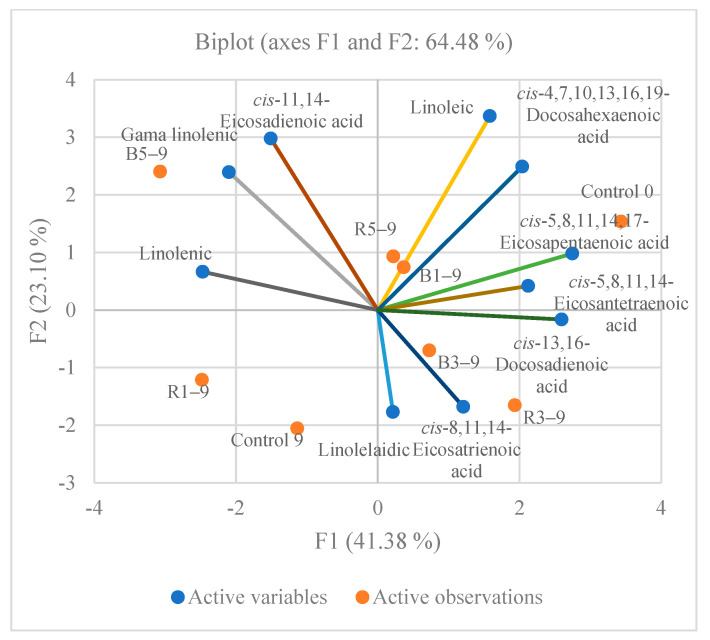
Principal component analysis (PCA) for polyunsaturated fatty acids of meat patties with different berries pomace additives stored for nine days (Control 0—meat patties without additives before storage; Control 9—without additives stored for 9 days); R1–9 and B1–9—with 1% of raspberry and blackberry pomace additives after 9 days of storage; R3–9 and B3–9—with 3% of raspberry and blackberry pomace additives after 9 days of storage; R5–9 and B5–9—with 5% of raspberry and blackberry pomace additives after 9 days of storage).

Principal component analysis (PCA) was performed to evaluate the relationships between the usage of berry pomace and the content of saturated, monounsaturated, and polyunsaturated acids in meat patties whereas all of the patties with additives were well separated in the PCA map (Figure 3, Figure 4 and Figure 5). The first two components (PCs) were associated with eigenvalues higher than one and explained 28.38% and 24.57% of the total variance for saturated, 33.80%, and 29.98% for the monounsaturated and 41.38% and 23.10% for the polyunsaturated acids (Figure 3, Figure 4 and Figure 5). As can be seen in Figure 3 for the saturated fatty acids, a separation of the samples clearly occurs, based on the addition of the berry pomace. All of the samples with the blackberry pomace, except those of 5% concentration, were distributed at negative PC2 values, while all of the control samples and samples with the raspberry pomace were situated at positive PC2 values. The control beef patties were closely related to the pentadecanic, stearic, and tricosanic fatty acids. The palmitic and stearic fatty acids were the predominant saturated fatty acids in the beef patties. A similar fatty acids profile in beef was described by Ganhão et al. [51]. The palmitic acid (C16:0) content after storage in the control beef patties was 1.76 times higher than before storage. An increase in the palmitic acid content was observed in goose meat refrigerated at 1 and 4 °C for 7 and 11 days [52]. Meanwhile the stearic acid content decreased with the increasing addition of the berry pomace into the beef patties after storage. In the beef patties with raspberry pomace after 9 days of storage, the stearic acid content decreased by 1.40 times, while with the blackberry—1.14. The beef patties with blackberry pomace had a high content of butyric acid compared with the patties of other formulations. The butyric acid content decreased with the increasing addition of pomace. The amount of tetracosanoic acid in the beef patties after storage compared with the control and the patties with blackberry pomace differed overall, regardless of the amount of blackberry additive, by 49.87 times. The blackberry addition increased the amount of tetracosanoic acid in the meat.

Figure 4 shows that the first factor (PC1) was highly and positively correlated with palmitoleic, erucic, myristoleic, *cis*-10-heptadecanic, and *cis*-10-pentadecenic fatty acids, and the highest contents were in the control beef patties before the storage. The second factor (PC2) is highly and positively related to nervonic fatty acid and the control beef patties after the storage, as well as with raspberry pomace, while negatively with eicosanoid fatty acid and the patties with the blackberry pomace. Monounsaturated fatty acids were predominant in the beef meat, with *cis*-9-oleic being the most abundant fatty acid in all of the meat samples. The *trans*-9-elaidic fatty acid content decreased in the meat patties after the storage and the main decrease was observed in the patties with the blackberry addition, compared with the control beef patties, by 1.26 times.

The *cis*-10-pentadecenic acid (C15:1) content in the control beef patties after the nine days of storage decreased by 1.8 times, the *cis*-10-heptadecanic acid (C17:1) by 1.7 times, and the *cis*-9-oleic acid (C18:1) by three percentage units. The content of the polyunsaturated fatty acids differed with the different amounts of pomace and the storage time of the beef patties. The principal component analysis showed that the higher content of *cis*-5,8,11,14,17-eicosapentaenoic acid, *cis*-4,7,10,13,16,19-docosahexaenoic acid, and *cis*-5,8,11,14-eicosantetraenoic acid was associated with the control beef patties before storage, while the gamma-linolenic acid was closely related with the beef patties with 5% of blackberry pomace after the storage (Figure 5). The linoleic (C18:2) and eicosatetraenoic (C20:4) fatty acids, in the control beef patties after storage, decreased twice, while the eicosapentaenoic (C20:5) acid content decreased by 1.4 percent units. In the goose muscle refrigerated for 11 days at 1 °C and 4 °C, the observed downward tendency in the PUFA content was mainly due to the decrease in the content of linoleic acid, linolenic acid, and arachidonic acid [52]. The research data show that the linoleic acid content in the control beef patties after storage for 9 days decreased by 1.93 times, while with the addition of 5% of raspberry pomace it decreased by 1.19, and with the addition of 5% of blackberry pomace—by 1.13 times. The linolenic acid content during storage increased in all of the formulation patties. The total amount of PUFA in the control beef patties before storage consisted of 13.51%, while after 9 storage days it was 10.12% in control patties; with the addition of 5% of raspberry pomace—9.87%, and with the addition of 5% of blackberry pomace—11.74%.

**Figure 6:**
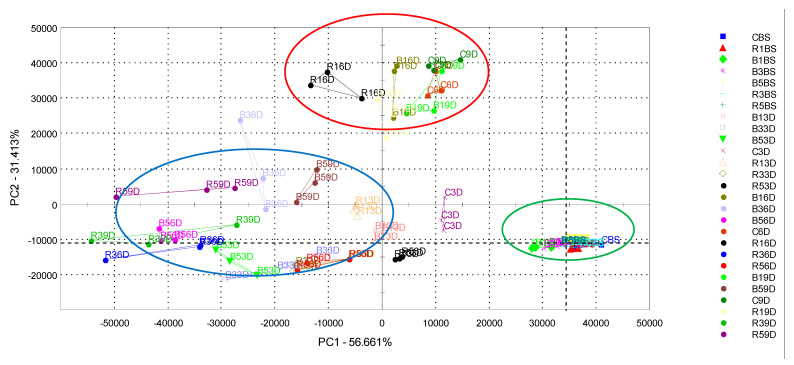
Principal component analysis (PCA) for volatile compounds of meat patties with different berries pomace additives stored for nine days (CBS—meat patties without additives before storage; C3D, C6D, C9D—without additives stored for 3, 6, 9 days; R1BS, B1BS, R3BS, B3BS, R5BS, B5BS—meat patties with 1, 3 and 5% of pomace additives before storage; R13D, B13D, R16D, B16D, R19D, B19D—with 1% of raspberry and blackberry pomace additives after 3, 6, 9 days of storage; R33D, B33D, R36D, B36D, R39D, B39D—with 3% of raspberry and blackberry pomace additives after 3, 6, 9 days of storage; R53D, B53D, R56D, B56D, R59D, B59D—with 5% of raspberry and blackberry pomace additives after 3, 6, 9 days of storage).

Additionally, in order to examine a possible grouping of the beef patties’ samples according to the aroma and used pomace, and storage time, a PCA was carried out and the obtained results are presented in Figure 6.

The first two components (PCs) explained 56.66% and 31.41% of the total variance of the data. As can be seen in Figure 6, the separation of the samples clearly occurs, based on the amount of pomace additive and storage time. Most of the samples before storage—the control samples, as well as the samples with different amounts of berry pomace—were situated at the positive PC1 values (marked in green color) (Figure S1), while the control beef patties and the patties with the addition of 1% of raspberry and blackberry pomace stored for 6 and 9 days were at the positive PC2 values (marked in red color) (Figure S2). The aroma profile was similar and situated at the negative PC1 values of beef patties with the addition of 3 and 5% of berry pomace stored for 3, 6, and 9 days (marked in blue) (Figure S3). The PCA analysis clearly shows that the berry pomace has a positive influence on the beef aroma.

## 4. Conclusions

The current study confirmed that the berry pomace shows great potential for usage in meat patties formulations. In summary, the berry pomace additive contributes to the reduction in the lipid oxidation and the changes of aroma profile, as well as the overall quality of the beef patties. Furthermore, at TBARS values of 2.0, the meat is considered to be unacceptable because of the off-flavors that are detectable. The beef patties stored for 6 and 9 days based on this indicator should be considered unsuitable for human consumption, while the pomace additive allows for a prolongation of the refrigerated storage of the product. VC analysis showed the difference in the aroma profile of the beef patties depending on the storage time and amount of pomace. Clear parallels can be drawn between the TBARS level and the aroma profile of the patties. In addition, this research will contribute to the usage of agro-food by-products in a more sustainable manner. Further studies are needed to raise awareness of the effect of the direct use of pomace additives.

## Figures and Tables

**Table 1:** Total phenols content and anthocyanins in berry pomace, mg 100 g^−1^ GAE and C3GE, DM.

Berry Pomace	Total Phenols	Total Anthocyanins
Raspberry	461.33 ^b^	184.07 ^b^
Blackberry	431.33 ^a^	24.45 ^a^

^a,b^ Values within a column with different superscripts differ significantly at *p* ≤ 0.05.

**Table 2:** Natural mass loss of beef patties stored for nine days, %.

Storage Days	Without Pomace	Raspberry Pomace			Blackberry Pomace		
		1%	3%	5%	1%	3%	5%
3	1.68 ^c^	0.05 ^f^	0.09 ^e,f^	0.07 ^f^	0.05 ^f^	0.05 ^f^	0.13 ^e,f^
6	2.10 ^b^	0.11 ^e,f^	0.19 ^e,f^	0.13 ^e,f^	0.11 ^e,f^	0.10 ^e,f^	0.22 ^d,e,f^
9	3.37 ^a^	0.16 ^e,f^	0.26 ^d,e^	0.20 ^e,f^	0.16 ^e,f^	0.15 ^e,f^	0.26 ^d,e^

^a,b,c,d,e,f^ Values within a columns and rows with different superscripts differ significantly at *p* ≤ 0.05.

**Table 3:** Beef patties pH changes during storage.

Storage Days	Without Pomace	Raspberry Pomace			Blackberry Pomace		
		1%	3%	5%	1%	3%	5%
0	5.41 ^c^	-	-	-	-	-	-
3	5.42 ^c^	5.13 ^f^	4.84 ^i^	4.68 ^j^	5.15 ^f^	5.63 ^a^	5.51 ^b^
6	5.41 ^c^	5.38 ^c^	4.94 ^h^	4.82 ^i^	5.25 ^e^	5.02 ^g^	4.95 ^h^
9	5.44 ^c^	5.31 ^d^	4.83 ^i^	4.68 ^j^	5.38 ^c^	5.04 ^g^	4.82 ^i^

^a,b,c,e,f,g,h,i,j^ Values within a columns and rows with different superscripts differ significantly at *p* ≤ 0.05.

**Table 4:** Correlation coefficient between beef patties color parameters and TBARS.

Color	Control Beef Patties	Beef Patties with Raspberry Pomace	Beef Patties with Blackberry Pomace
L*	−0.270	−0.733	0.293
a*	−0.900	−0.700	0.291
b*	−0.840	−0.745	−0.163

## Data Availability

Data are contained within the article.

## Supplementary Materials

The following supporting information can be downloaded at: https://www.mdpi.com/article/10.3390/foods11152180/s1, Figure S1: Principal component analysis (PCA) for volatile compounds of meat patties with different berries pomace additives stored for nine days (CBS—meat patties without additives before storage; C3D, C6D, C9D—without additives stored for 3, 6, 9 days; R1BS, B1BS, R3BS, B3BS, R5BS, B5BS—meat patties with 1, 3 and 5% of pomace additives before storage; R13D, B13D, R16D, B16D, R19D, B19D—with 1% of raspberry and blackberry pomace additives after 3, 6, 9 days of storage; R33D, B33D, R36D, B36D, R39D, B39D—with 3% of raspberry and blackberry pomace additives after 3, 6, 9 days of storage; R53D, B53D, R56D, B56D, R59D—with 5% of raspberry and blackberry pomace additives after 3, 6, 9 days of storage); Figure S2. Principal component analysis (PCA) for volatile compounds of meat patties with different berries pomace additives stored for nine days (CBS—meat patties without additives before storage; C3D, C6D, C9D—without additives stored for 3, 6, 9 days; R1BS, B1BS, R3BS, B3BS, R5BS, B5BS—meat patties with 1, 3 and 5% of pomace additives before storage; R13D, B13D, R16D, B16D, R19D, B19D—with 1% of raspberry and blackberry pomace additives after 3, 6, 9 days of storage; R33D, B33D, R36D, B36D, R39D, B39D—with 3% of raspberry and blackberry pomace additives after 3, 6, 9 days of storage; R53D, B53D, R56D, B56D, R59D—with 5% of raspberry and blackberry pomace additives after 3, 6, 9 days of storage); Figure S3. Principal component analysis (PCA) for volatile compounds of meat patties with different berries pomace additives stored for nine days (CBS—meat patties without additives before storage; C3D, C6D, C9D—without additives stored for 3, 6, 9 days; R1BS, B1BS, R3BS, B3BS, R5BS, B5BS —meat patties with 1, 3 and 5% of pomace additives before storage; R13D, B13D, R16D, B16D, R19D, B19D—with 1% of raspberry and blackberry pomace additives after 3, 6, 9 days of storage; R33D, B33D, R36D, B36D, R39D, B39D—with 3% of raspberry and blackberry pomace additives after 3, 6, 9 days of storage; R53D, B53D, R56D, B56D, R59D—with 5% of raspberry and blackberry pomace additives after 3, 6, 9 days of storage).
