# Supplementary material for: The Effect of Berry Pomace on Quality Changes of Beef Patties during Refrigerated Storage
Source: Foods. 2022 Jul 22;11(15):2180. doi: 10.3390/foods11152180 (PMC9331956; doi:10.3390/foods11152180)
Supplement: Supplementary file 1 [file foods-11-02180-s001.zip › foods-1792628-supplementary.pdf]

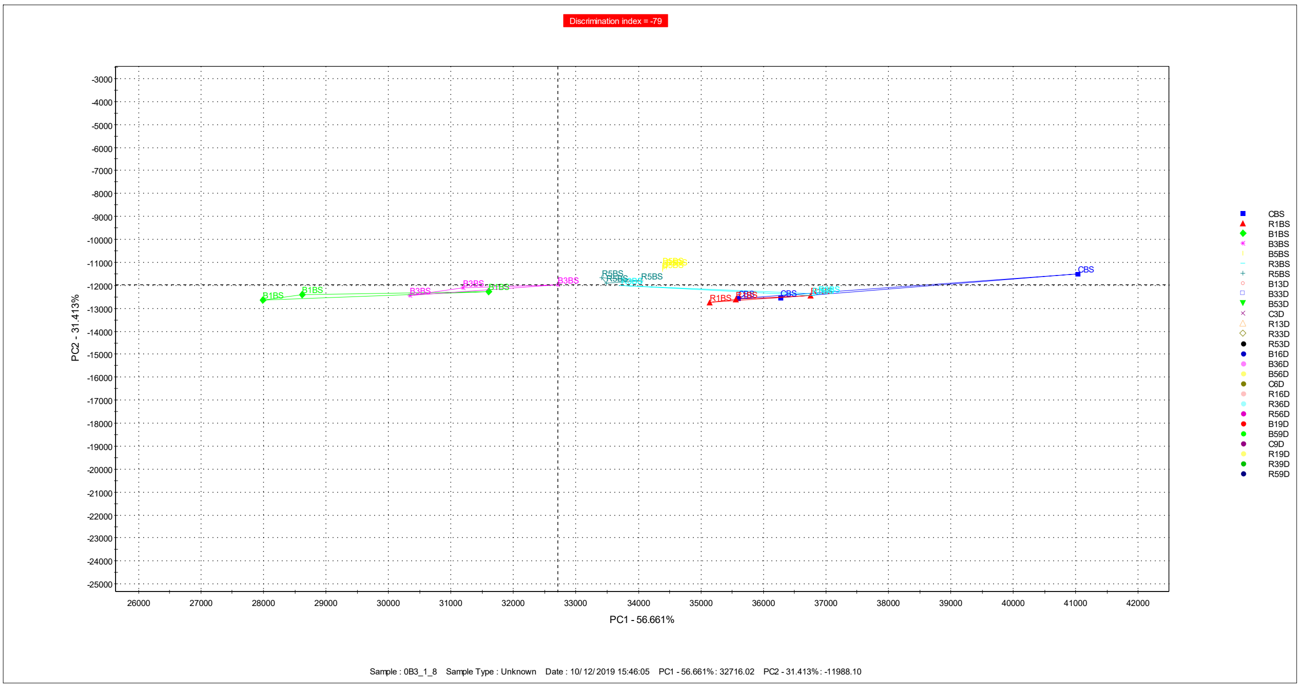

Figure S1. Principal component analysis (PCA) for volatile compounds of meat patties with different berries pomace additives stored for nine days (CBS—meat patties without additives before storage; C3D, C6D, C9D—without additives stored for 3, 6, 9 days; R1BS, B1BS, R3BS,B3BS, R5BS, B5BS —meat patties with 1, 3 and 5% of pomace additives before storage; R13D, B13D, R16D, B16D, R19D, B19D -–with 1% of raspberry and blackberry pomace additives after 3, 6, 9 days of storage; R33D, B33D, R36D, B36D, R39D, B39D—with 3% of raspberry and blackberry pomace additives after 3, 6, 9 days of storage; R53D, B53D, R56D, B56D, R59D,—with 5% of raspberry and blackberry pomace additives after 3, 6, 9 days of storage).

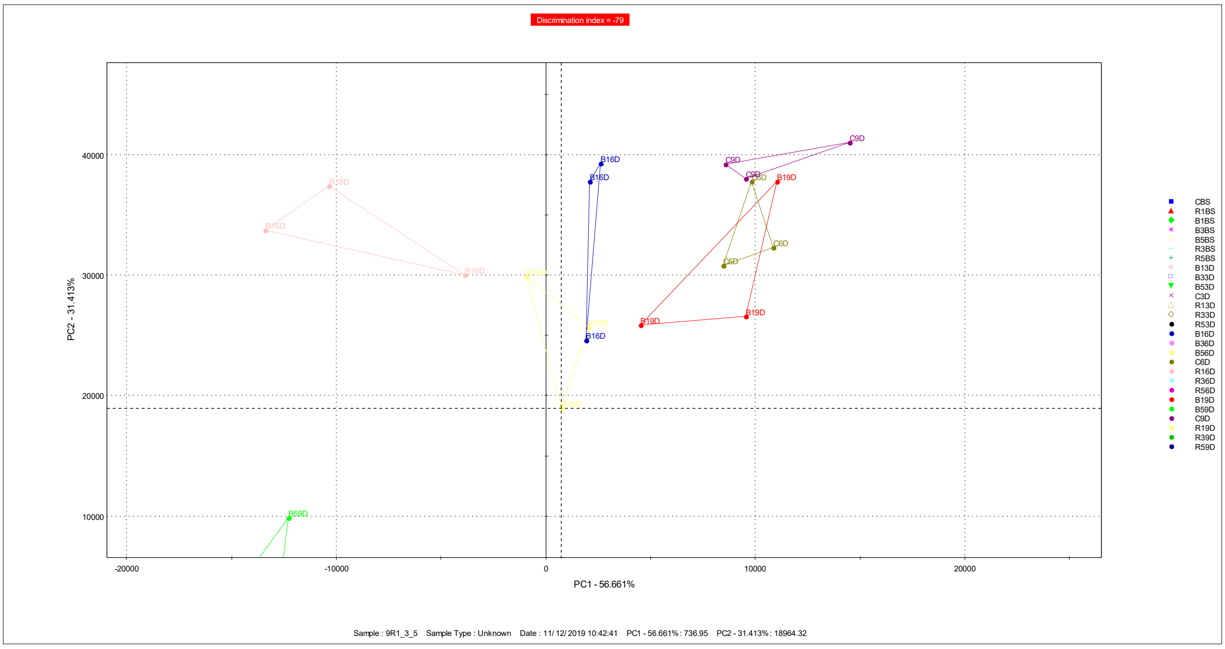

Figure S2. Principal component analysis (PCA) for volatile compounds of meat patties with different berries pomace additives stored for nine days (CBS—meat patties without additives before storage; C3D, C6D, C9D—without additives stored for 3, 6, 9 days; R1BS, B1BS, R3BS,B3BS, R5BS, B5BS —meat patties with 1, 3 and 5% of pomace additives before storage; R13D, B13D,

R16D, B16D, R19D, B19D -–with 1% of raspberry and blackberry pomace additives after 3, 6, 9 days of storage; R33D, B33D, R36D, B36D, R39D, B39D—with 3% of raspberry and blackberry pomace additives after 3, 6, 9 days of storage; R53D, B53D, R56D, B56D, R59D,—with 5% of raspberry and blackberry pomace additives after 3, 6, 9 days of storage).

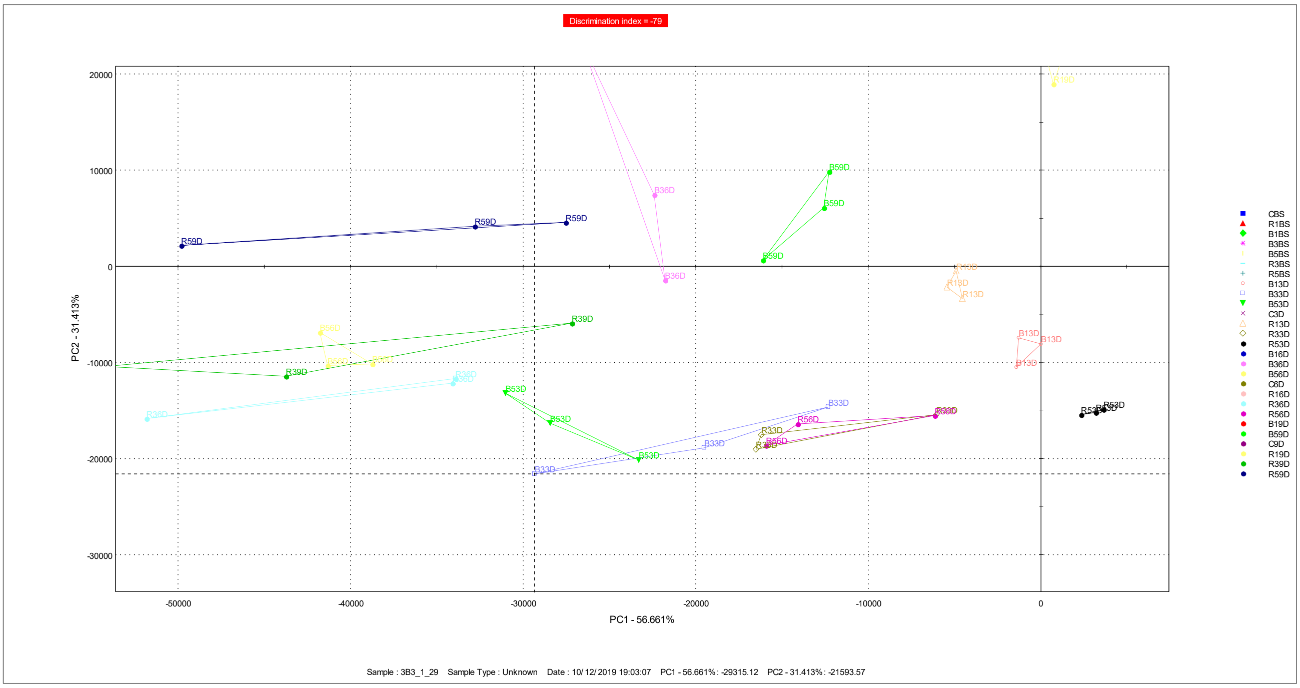

Figure S3. Principal component analysis (PCA) for volatile compounds of meat patties with different berries pomace additives stored for nine days (CBS—meat patties without additives before storage; C3D, C6D, C9D—without additives stored for 3, 6, 9 days; R1BS, B1BS, R3BS,B3BS, R5BS, B5BS —meat patties with 1, 3 and 5% of pomace additives before storage; R13D, B13D, R16D, B16D, R19D, B19D -–with 1% of raspberry and blackberry pomace additives after 3, 6, 9 days of storage; R33D, B33D, R36D, B36D, R39D, B39D—with 3% of raspberry and blackberry pomace additives after 3, 6, 9 days of storage; R53D, B53D, R56D, B56D, R59D,—with 5% of raspberry and blackberry pomace additives after 3, 6, 9 days of storage).
